# The complete chloroplast genome sequence of the North American sclerophyllous evergreen shrub, *Quercus turbinella* (Fagaceae)

**DOI:** 10.1080/23802359.2024.2305398

**Published:** 2024-01-18

**Authors:** Amarsanaa Gantsetseg, Eun-Kyeong Han, Jung-Hyun Lee

**Affiliations:** Department of Biology Education, Chonnam National University, Gwangju, Republic of Korea

**Keywords:** Chloroplast genome, *Quercus turbinella*, phylogenetic analysis

## Abstract

*Quercus turbinella* (section *Quercus*; Fagaceae) is an evergreen shrub characteristic in central Arizona and it concerns one of the most abundant and economically important genera of *Quercus* in the Northern Hemisphere. Here, we have sequenced the complete chloroplast genome to provide insight into the phylogenetic relationship of *Q. turbinella*. The whole genome is 161,208 bp in length with two inverted repeat regions of 25,827 bp each, which separate a large single-copy region of 90,552 bp and a small single-copy region of 19,002 bp. A total of 136 genes were annotated, including 88 protein-coding genes, eight ribosomal RNAs, and 40 transfer RNAs. The result of the maximum-likelihood phylogenetic analysis strongly suggested that *Quercus turbinella* had a close relationship to *Quercus macrocarpa* with strong bootstrap support.

## Introduction

Around 56 million years ago, oak trees first appeared (Kremer and Hipp 2019). The *Quercus* L. (Fagaceae) genus, commonly known as oaks, encompasses over 450 species, establishing itself as a dominant presence in diverse ecosystems across the Northern Hemisphere and holding considerable economic and ecological importance (Manos et al. 1999). To gain a better understanding of the evolutionary significance of oak species, it is essential to explore their diverse ecological contexts (Han et al. 2023). Currently, available data provide valuable insights, but there is still much to uncover regarding the evolution of oak species. *Quercus turbinella* Greene 1889 is a sclerophyllous evergreen shrub that thrives in the arid regions of the western United States and northern Mexico (Tucker 1953; Holmgren et al. 2011), especially in chaparral ecosystems in central Arizona, where it serves as the dominant shrub, adapting to a range of sites and exposures (Pase 1969). In this study, we report the complete chloroplast genome of *Q. turbinella*, which will serve as a fundamental resource for understanding the evolution and phylogeny of oaks.

## Materials and methods

### Plant materials and DNA extraction

**Figure 1.**
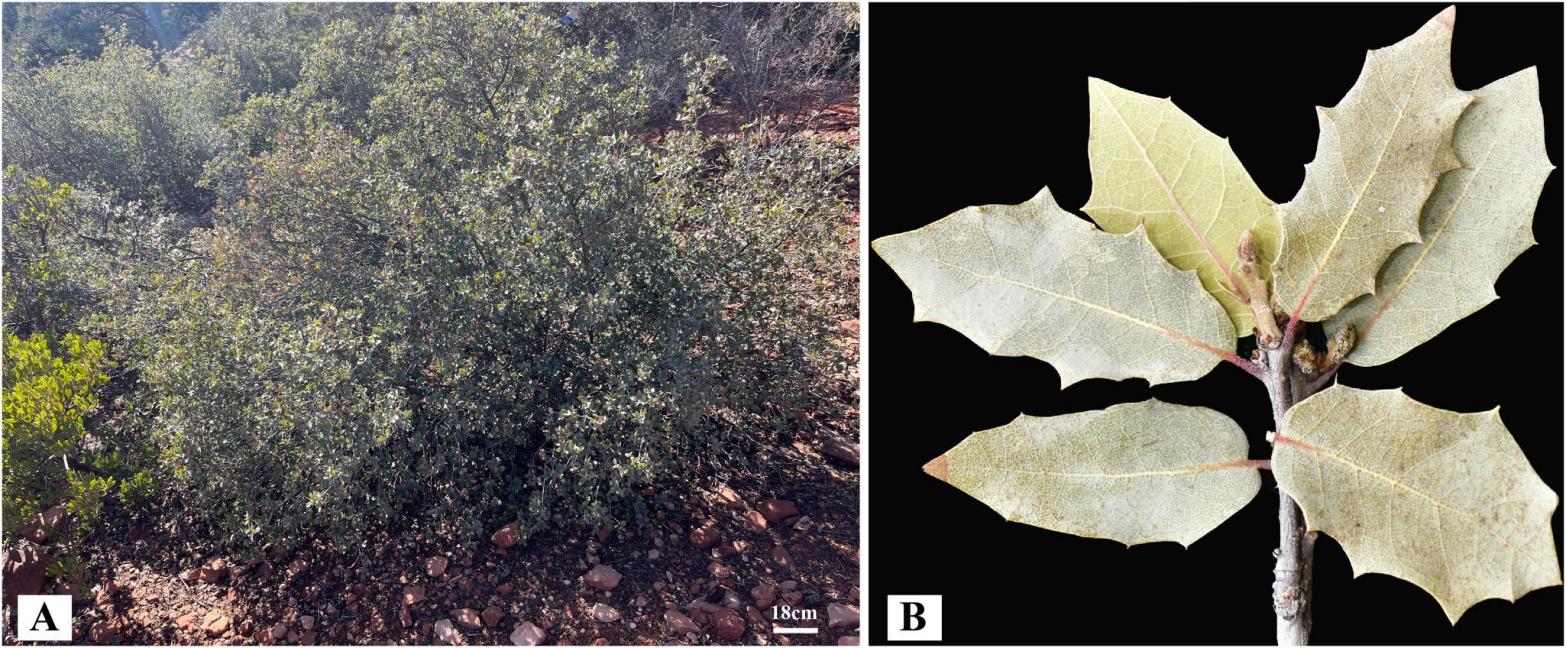
The habit (A) and morphological characteristics (B) of *Quercus turbinella. Q. turbinella* is an evergreen desert scrub oak, that is predominantly found in pinyon-juniper forests and desert slopes. Bark light gray or brown; twigs brown to gray, 1–3 mm diam., usually tomentose or sometimes glabrous, becoming glabrate. Leaf-blade elliptic or ovate, thick, leathery, base cordate or rounded, margins planar or slightly crisped-undulate, apex acute or obtuse; abaxial surface yellow or reddish, usually glaucous, adaxial surface grayish, glaucous, or yellowish glandular, sparsely and minutely stellate-pubescent or glabrous. Photos taken by professor Jung-Hyun Lee in Thunder Mountain Trail, Sedona, AZ, United States, February 2023, without any copyright issues.

Fresh leaves of *Q. turbinella* were collected from Thunder Mountain Trail, Sedona, AZ, United States (34°52′40.1ʺ N, 111°48′34.9ʺ W; Figure 1) and dried with silica gel. The voucher specimen and its DNA sample were deposited at the herbarium of the Department of Biology Education, Chonnam National University (Prof. Jung-Hyun Lee, E-mail: quercus@jnu.ac.kr) under the voucher number Lee-AZ20230218. Total genomic DNA was extracted from dried leaves using a DNeasy Plant Mini Kit (Qiagen, Seoul, South Korea).

### Plastome sequencing, assembly and annotation

The DNA library was constructed with the TruSeq Nano DNA Kit (Macrogen, Seoul, South Korea) and sequenced on the Illumina HiSeq X platform (Illumina, San Diego, CA) following manufacturer protocol. Sequencing generated 54,033,292 raw paired-end reads (150 bp) that were then assembled in NOVOPlasty 4.1 (Dierckxsens et al. 2017). The selected seed sequence was complete chloroplast genome of *Q. glaucoides* (Yang et al. 2021; MG678003). Checking the assembled plastome, 2,850,980 reads were mapped in Geneious Prime 2022.2.2 (Kearse et al. 2012), resulting in a coverage of 4111× (Figure S1). Additionally, the plastome was annotated in Geneious and manually corrected for start and stop codons, as well as intron/exon boundaries. The complete chloroplast genome sequence of *Q. turbinella* was submitted to GenBank of the National Center for Biotechnology Information (NCBI, accession number OQ835463). The gene graphical map of the chloroplast genome structure and schematic maps of the cis-splicing genes and trans-splicing genes were illustrated using the CPGView (http://www.1kmpg.cn/cpgview; Liu et al. 2023).

### Phylogenetic analysis

To construct a maximum-likelihood (ML) tree for the *Q. turbinella* phylogenetic relationship, the chloroplast genome of 18 *Quercus* species (seven species from subgenus *Cerris* and 11 species from subgenus *Quercus*) were downloaded from NCBI. The outgroup used was *Fagus engleriana* (KX852398) from subgenus *Fagus*. Sequence alignment was performed by multiple alignment using MAFFT (Katoh and Toh 2010). ML tree was constructed using IQ-TREE web server (http://iqtree.cibiv.univie.ac.at/) with 1000 ultra-fast bootstrap replicates and following a best-fit model that was K3Pu + F + I + G4 (Nguyen et al. 2015; Hoang et al. 2017; Kalyaanamoorthy et al. 2017).

## Results and discussion

### Structural characteristics

**Figure 2.**
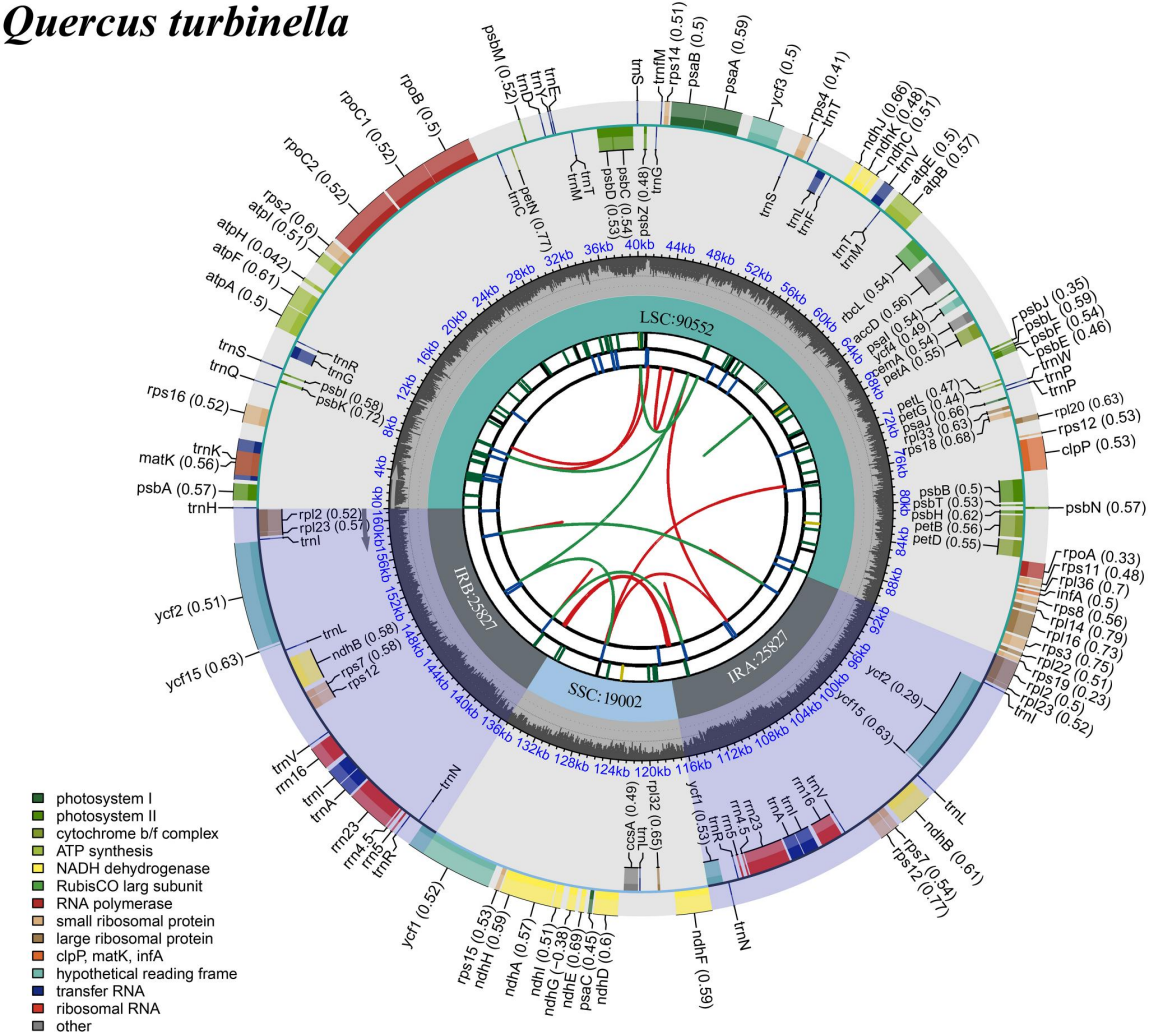
Schematic map of overall features of the chloroplast genome of *Quercus turbinella.* The circular map of the chloroplast genome was generated using CPGview (Liu et al. 2023). The map comprised seven circles, each depicting different features. Starting from the center and moving outwards, the first circle illustrated distributed repeats with red arcs indicating the forward direction and green arcs indicating the reverse direction. The second circle depicted tandem repeats through short bars. The third circle displayed microsatellite sequences represented by short bars. The fourth circle indicated the sizes of the LSC and SSC regions. The fifth circle represented the IRA and IRB regions. The sixth circle demonstrated the GC contents across the plastome. Lastly, the seventh circle displayed genes distinguished by different colors corresponding to their functional groups.

The length of the assembled *Q. turbinella* complete chloroplast genome was 161,208 bp with the typical conserved quadripartite structure, with a large single-copy (LSC) region of 90,552 bp, a small single-copy (SSC) region of 19,002 bp, and two inverted repeat (IR) regions of 25,827 bp (Figure 2). A total of 136 functional genes were identified, including 88 protein-coding genes (PCGs), eight ribosomal RNA genes (rRNAs), and 40 transfer RNA genes (tRNAs). Of these genes, 15 genes (*rps16*, *atpF*, *rpoC1*, *petB*, *petD*, *rpl16*, *rpl2*, *ndhB*, *ndhA*, *trnK-UUU*, *trnG-GCC*, *trnL-UAA*, *trnV-UAC*, *trnl-GAU*, and *trnA-UGC*) had one intron and three genes (*rps12*, *clpP*, and *ycf3*) contained two introns. Totally, the genome contained 13 *cis*-splicing genes and one *trans*-splicing gene, which is *rps12* (Figure S2). In the IR regions, 17 genes were duplicated including six PCGs (*rpl2*, *rpl23*, *ycf2*, *ycf15*, *ndhB*, and *rps7*), four rRNAs (4.5S, 5S, 16S, and 23S rRNA), and seven tRNAs (*trnI-CAU*, *trnL-CAA*, *trnV-GAC*, *trnI-GAU*, *trnA-UGC*, *trnR-ACG*, and *trnN-GUU*). The *ycf1* gene was located at the boundaries of IRa/SSC and IRb/SSC respectively. The total GC content in the chloroplast DNA was 36.8%, and in the LSC, SSC, and IR regions were 34.7%, 31.0%, and 42.8%, respectively. Gene content and order are similar to those of *Q. macrocapra* and *Q. dentata* (Pang et al. 2019).

### Phylogenetic analyses

**Figure 3.**
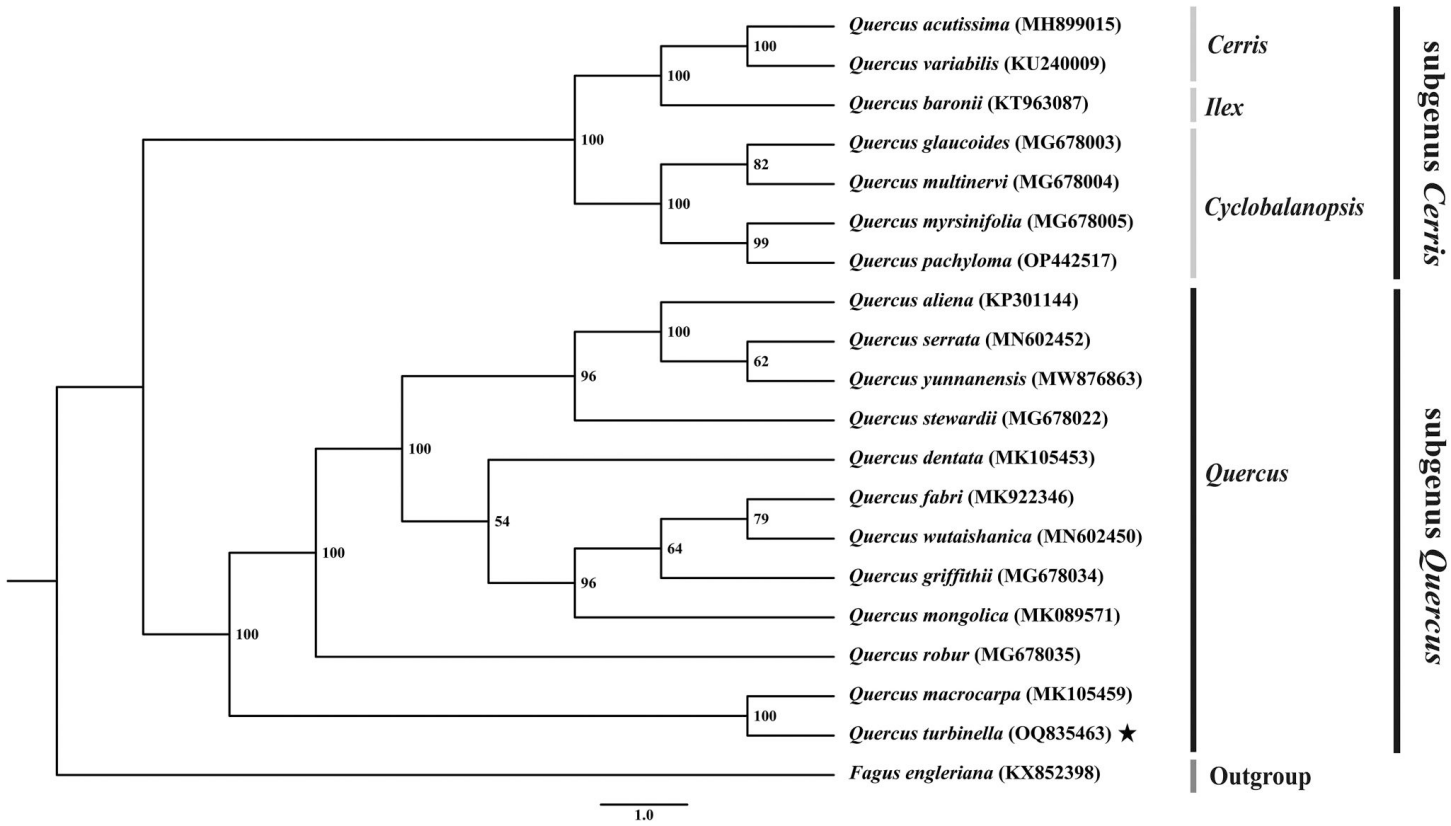
Construction of phylogenetic tree (ML) based on complete chloroplast genome of *Q. turbinella* and 18 other *Quercus* species. The sequence of *Fagus engleriana* was used as the outgroup. The following sequences were used: *Q. acutissima* MH899015 (Zhang et al. 2020), *Q. variabilis* KU240009 (Yang et al. 2016), *Q. baronii* KT963087 (Yang et al. 2015), *Q. glaucoides* MG678003 (Yang et al. 2021), *Q. multinervi* MG678004 (Yang et al. 2021), *Q. myrsinifolia* MG678005 (Yang et al. 2021), *Q. pachyloma* OP442517, *Q. aliena* KP301144, *Q. serrata* MN602452 (Liu et al. 2021), *Q. yunnanensis* MW876863 (Yang 2021), *Q. stewardii* MG678022 (Yang et al. 2021), *Q. dentata* MK105455, *Q. fabri* MK922346 (Xu et al. 2019), *Q. wutaishanica* MN602450 (Liu et al. 2021), *Q. griffithii* MG678034 (Yang et al. 2021)*, Q. mongolica* MK089571, *Q. robur* MG678035 (Yang et al. 2021), *Q. macrocarpa* MK105459 (Pang et al. 2019), *Fagus engleriana* KX852398 (Yang et al. 2018). The numbers above the nodes indicate bootstrap values with 1,000 replicates.

Based on the complete chloroplast genome of *Q. turbinella* and related taxa, the ML phylogenetic tree revealed two major clades within the genus *Quercus*. The clade one consists of the subgenus *Cerris*, while the clade two is composed of the subgenus *Quercus*. Notably, the phylogenetic tree demonstrates that *Q. turbinella* is closely related to *Q. macrocarpa*, supported by a 100% bootstrap value (Figure 3). Subgenus *Cerris* (comprising *Cerris*, *Ilex*, and *Cyclobalanopsis*) forms a paraphyletic clade to the subgenus *Quercus*.

## Conclusions

This study represents the first assembly of the chloroplast genome sequence of *Q. turbinella* and the subsequent annotation of its structure. The complete chloroplast (CP) genome data can be valuable for evaluating the genetic diversity, population structure, and phylogeographic history of this species and it will infer the molecular identification of this species. Moreover, the newly generated phylogenetic data can be used in future investigations into the evolutionary dynamics of the subgenus *Quercus* and *Cerris*.

## Ethical approval

The study species used in this research do not belong to the Endangered, Threatened, and Rare plants of the Natural Resources Conservation Service (United States Department of Agriculture) and list of native southeastern Arizona wildflowers. Additionally, Plant materials for this research were not collected from protected areas in Arizona, U.S. Therefore, this study does not need ethical approval or permission to collect the sample. The authors committed no ethical or legal violations during the acquisition of the study materials and the execution of the research.

## Supplementary Material

Supplemental Material

## Data Availability

The genome sequence data of *Quercus turbinella* that support the findings of this study are openly available in GenBank of NCBI at (https://www.ncbi.nlm.nih.gov/) under the accession no. OQ835463. The associated BioProject, SRA, and Bio-Sample numbers are PRJNA957934, SRR24235292, and SAMN34273566, respectively.
