# Supplementary material for: The complete chloroplast genome sequence of the North American sclerophyllous evergreen shrub, Quercus turbinella (Fagaceae)
Source: Mitochondrial DNA B Resour. 2024 Jan 18;9(1):123–7. doi: 10.1080/23802359.2024.2305398 (PMC10802804; doi:10.1080/23802359.2024.2305398)
Supplement: Supplemental Material [file TMDN_A_2305398_SM9860.docx]

Figure captions

Figure S1. Overall coverage depth of the chloroplast genome assembly of *Quercus turbinella*.

Figure S2. Schematic map of the cis and trans splicing genes in the chloroplast genome of *Quercus turbinella*.


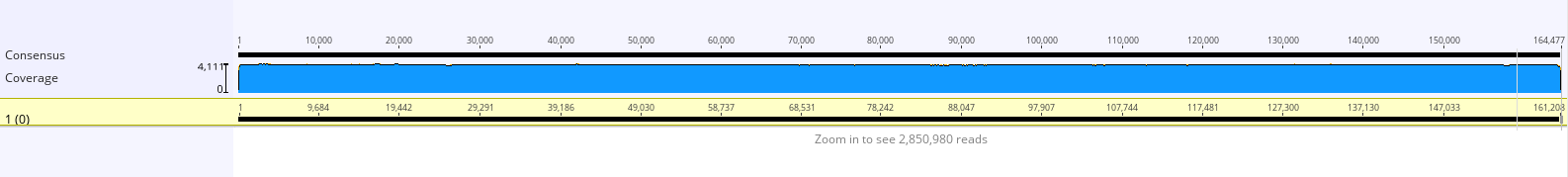


Figure S1. Using Geneious Prime, the DNA-Seq data were aligned to the whole chloroplast genome to generate a figure representing the overall coverage depth of the chloroplast genome assembly of *Quercus turbinella*. The height of the blue graph indicates the number of sequences at each location.


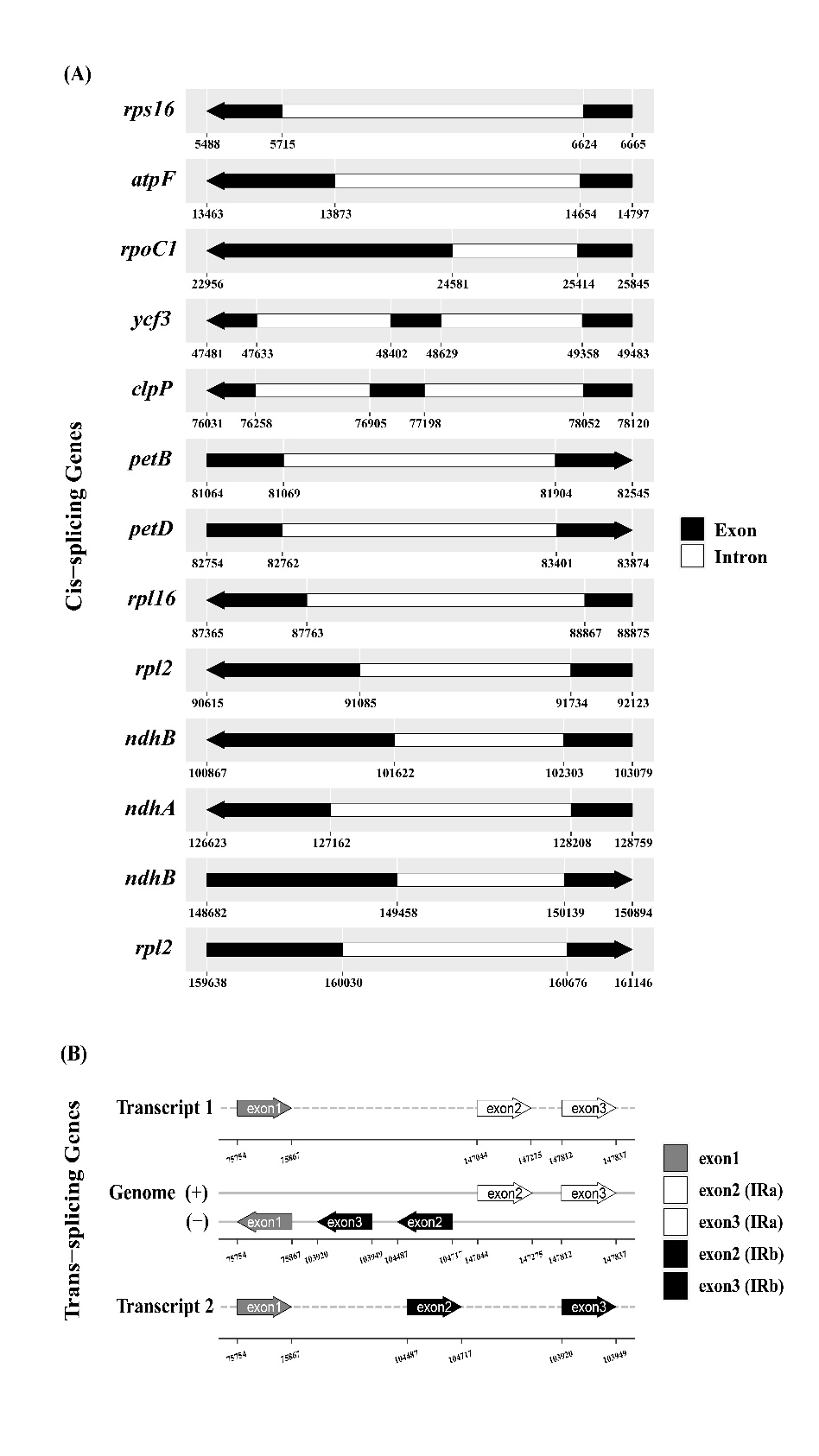


Figure S2. Schematic map of the cis-splicing genes (A) and trans-splicing gene rps12 (B) in the chloroplast genome of *Quercus turbinella*. Using CPGview, a map was generated to display the exons in black and the introns in white. The arrow indicates the sense direction of the gene.
